# Mitochondrial DNA diversity of the Sardinian local cattle stock

**DOI:** 10.1038/s41598-022-06420-3

**Published:** 2022-02-15

**Authors:** Elena Petretto, Maria Luisa Dettori, Michele Pazzola, Fabio Manca, Marcel Amills, Giuseppe Massimo Vacca

**Affiliations:** 1 Department of Veterinary Medicine, University of Sassari, Via Vienna 2, 07100 Sassari, Italy; 2 Departament de Ciència Animal i dels Aliments, Universitat Autònoma de Barcelona, 08193 Bellaterra, Spain; 3 Centre for Research in Agricultural Genomics (CRAG), CSIC-IRTA-UAB-UB, Campus Universitat Autònoma de Barcelona, 08193 Bellaterra, Spain

**Keywords:** DNA, Evolution, Genetics

## Abstract

The aim of this research was to characterize the genetic diversity of the Sarda (Sa, n = 131), Sardo Bruna (SB, n = 44) and Sardo Modicana (SM, n = 26) cattle breeds, reared in the island of Sardinia (Italy). A portion of the mitochondrial DNA hypervariable region was sequenced, in order to identify a potential signature of African introgression. The F_ST_ coefficients among populations ranged between 0.056 for Sa vs SB and 0.167 for SB vs SM. AMOVA analysis indicated there was a significant differentiation of the three breeds, although most of diversity was gathered at the within-breed level. The Median Joining Network of the Sardinian sequences showed a potential founder effect signature. A MJ network including Sardinian cattle plus African, Italian, Iberian and Asian sequences, revealed the presence of haplogroup T3, already detected in Sa cattle, and the presence of Hg T1 and Hg T1′2′3, in Sa and SB. The presence of a private haplotype belonging to haplogroup T1, which is characteristic of African taurine breeds, may be due to the introgression of Sardinian breeds with African cattle, either directly (most probable source: North African cattle) or indirectly (through a Mediterranean intermediary already introgressed with African blood).

## Introduction

Livestock breeding systems have experienced substantial changes during the twentieth century, mainly driven by mechanization, industrialization, and intensive selection. This process, which resulted in the adoption and diffusion throughout the world of highly selected cosmopolitan breeds^1^, led to an impressive improvement of productions and to a genetic homogenization of farmed animals caused by the progressive replacement of rustic local breeds by their cosmopolitan counterparts^2^. Local breeds are an important cultural legacy and they play a fundamental role in landscape maintenance, being a key insurance against unknown forthcomings such as climate change and disease outbreaks^3,4^. Local and autochthonous breeds have undergone natural selection during millenia resulting in an optimal adaptation to a specific milieu^5^. For all these reasons, local breeds should be preserved as an essential asset for sustainable farming in the future^6^. Part of these conservation efforts have been devoted to the genetic characterization of these irreplaceable animal resources^6^.

Sardinia (Italy) is a large and ancient island in the western Mediterranean Sea. Traditionally, sheep and goat farming have had an important impact in the rural economy of Sardinia^7,8^. In addition, three local cattle breeds are currently reared in Sardinia: the Sarda, the Sardo Bruna and the Sardo Modicana^9^. Sarda cattle are small sized, with high hardiness and resistance. They are perfectly adapted to the mountainous areas with arid soils in which they are typically raised. Historically, Sarda cattle provided milk, meat and labor to farmers, but during the 1880s, and for about fifty years, this breed was extensively crossed with bulls from the Brown breed, originary from Switzerland, with the aim of improving dairy traits. Moreover, Sarda cattle were also crossed with bulls from the Modicana breed, native to Sicily, with the goal of improving their work aptitude. Absorption crosses led to the transformation, in some areas of Sardinia, of the original Sarda cattle into two different populations i.e. Sardo Bruna, virtually equivalent to the Brown Swiss cattle, and Sardo Modicana, morphologically similar to the Sicilian Modicana. According to FAO^10^, the number of Sarda cattle reared in Sardinia lies close to 21,800 individuals, while the Sardo Bruna and the Sardo Modicana breeds are represented by 27,670 heads and 2.200 heads, respectively. The government of Sardinia has established a herd register for each breed and herd books are managed by the Italian Breeders Association^11^.

Decker et al*.*^12^ investigated the patterns of ancestry, divergence and admixture of cattle by genotyping 43,043 single nucleotide polymorphisms (SNP) in 1,543 bovines from 134 breeds with a worldwide distribution. One of the main conclusions of this work was that Iberian and Italian cattle had been introgressed with African blood^12^. Although the genome-wide diversity of the Sarda, Sardo Bruna and Sardo Modicana cattle has been characterized in previous studies^13,14^ and the complete mitochondrial genome of one Sarda cattle (GenBank EU177832) has been sequenced^15^, the potential African introgression of bovine breeds from Sardinia has never been explored in depth. In this regard, the analysis of mitochondrial data could be really useful because the T3 and T1 haplogroups are vastly predominant in Europe and Africa, respectively^16^. In the current work, we aimed to characterize the genetic diversity of the Sarda (Sa), Sardo Bruna (SB) and Sardo Modicana (SM) breeds through the partial sequencing of the mitochondrial DNA hypervariable region in order to identify a potential signature of African introgression.

## Results

About 616 bp of the mtDNA hypervariable region (GenBank V00654 was the reference sequence) were successfully sequenced in 201 female cattle from the island of Sardinia (supplementary Table S1). Alignment of 200 sequences corresponding to Sa, SB and SM cattle revealed the occurrence of 34 polymorphic sites and 32 haplotypes (supplementary Table S2), while overall haplotype diversity was 0.878 (Table 1). The highest haplotype number was observed in the Sa breed, with 22 haplotypes out of 131 sampled animals, and it was similar to the SB breed, which had 15 haplotypes out of 43 sampled animals (Table 1). In SM cattle we found only 5 haplotypes out of 26 sequences, but it should be kept in mind that all individuals came from the same sampling site (Milis).

**Table 1:** Distribution of mtDNA haplotypes in three local cattle breeds from Sardinia (616 bp mapping to the hypervariable region, range 15,792 – 69 of acc. no. V00654).

Parameters	Sarda	Sardo Bruna	Sardo Modicana	Overall	Italian Brown*	Modicana*	Maremmana*	Italian Podolian*
Sample size	131	43	26	200	34	33	62	91
Number of polymorphic sites	26	22	6	34	–	–	–	–
Tajima’s D	− 0.677 Ns	− 1.307 Ns	− 0.069	− 1.180 Ns	–	–	–	–
Nucleotide diversity	0.006	0.005	0.002	0.005	0.005	0.005	0.006	0.005
Number of haplotypes	22	15	5	32	–	–	–	–
Haplotype diversity	0.875	0.827	0.662	0.879	0.929	0.864	0.973	0.872
Detected haplogroups	T1 (22), T3 (104)	T1 (1), T3 (40)	T3 (26)	T1, T3	T1, T3	T1, T3	T1, T2	T1, T2
	T1′2′3 (5)	T1′2′3 (2)		T1′2′3	T5		T3	T3

*Di Lorenzo et al.^19^. The range of the mtDNA hypervariable region was 15,823–215. Ns, non significant.

**Figure 1:**
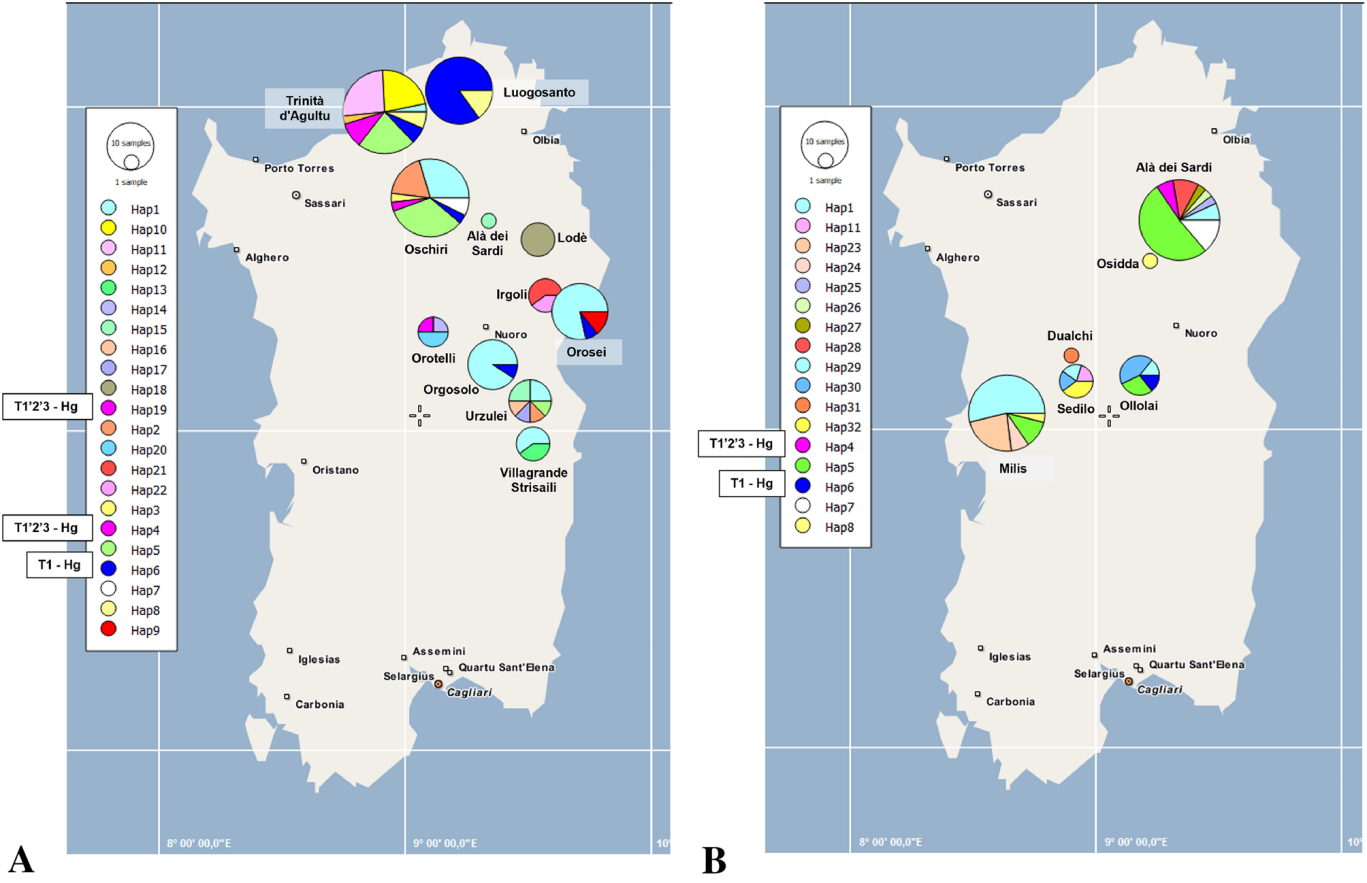
Geographic distribution of mtDNA haplotypes. The map was created by authors using the PopArt software, version 1.7 (http://popart.otago.ac.nz/index.shtml), (Leigh and Bryant, 2015). Pie chart slices are proportional to the frequency of the haplotypes. (**A**) Sampling sites and haplotypes found in the Sarda breed cattle. (**B**) Sampling sites and haplotypes segregating in the Sardo Bruna breed cattle, except for the Milis sampling site, where all animals were of the Sardo Modicana breed. Haplotypes belonging to the T1 haplogroup are indicated in blue (T1 – Hg), Haplotypes of the T1’2’3 haplogroup are indicated in pink (T1’2’3 – Hg), all the other haplotypes were represented with pastel colours, and belong to the T3 haplogroup.

Geographic distribution of haplotypes in the island of Sardinia is shown in Fig. 1. Different colours have been given to each haplotype. Moreover, each haplotype has been represented only once for each sampling site where it occurred, in order to avoid the overlapping of clusters. Sampling sites for the Sa breed are represented in Fig. 1A (eleven sites), while sampling sites for the SB (five sites) and SM (only one site, Milis) breeds are shown in Fig. 1B. The inspection of Fig. 1 evidences that there was not any geographic structure associated with the distribution of mtDNA haplotypes in Sardinia.

The F_ST_ coefficients among populations ranged between 0.056 for Sa vs SB and 0.167 for SB vs SM (Table 2). AMOVA analysis indicated there was a significant differentiation of the three breeds (between-populations component of variation of 7.99%) although most of diversity was gathered at the within-breed level (Table 3).

**Table 2:** F_ST_ values calculated with DnaSP based on mtDNA data from three Sardinian local cattle breeds.

	Sarda	Sardo Modicana
Sardo Modicana	0.136	
Sardo Bruna	0.056	0.167

**Table 3:** Analysis of molecular variance (AMOVA) based on mtDNA data from Sardinian cattle breeds.

Source of variation	d.f.	Sum of squares	Variance components	Percentage of variation
Among populations	2	17.659	0.14172 Va	7.99
Within populations	197	321.536	1.63216 Vb	92.01
Total	199	339.195	1.77388	
Fixation Index F_ST_: 0.07989		P-value = 0.00010		

**Figure 2:**
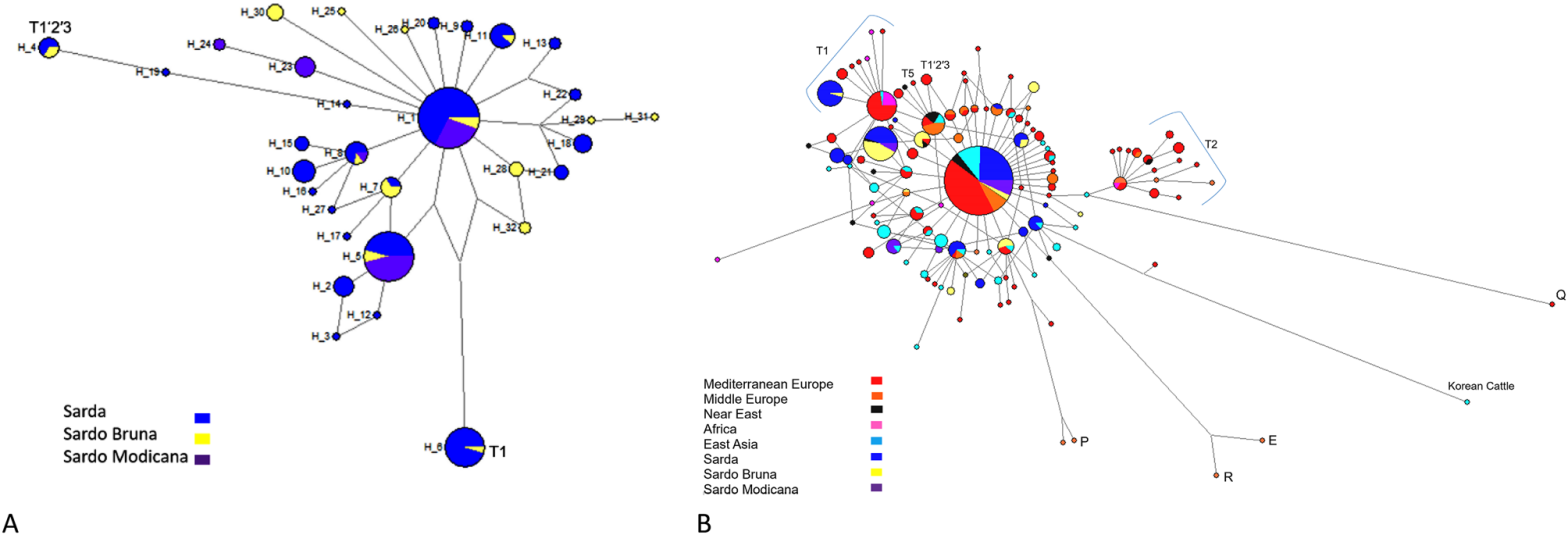
(**A**) Median Joining Network based on mtDNA hypervariable sequence haplotypes of 157 Sardinian cattle, including sequences from Sarda, Sardo Bruna and Sardo Modicana cattle breeds. T1 and T1,2,3 indicate the Haplogroup assignment. Aligned sequences were 616 bp long. (**B**) Median joining network based on mtDNA hypervariable sequence haplotypes of 586 sequences from 157 Sardinian cattle and European, Asian and African bovines retrieved from public databases and representative of all known mitochondrial haplogroups. Sequences have been trimmed to obtain an alignment of 487 bp. Mediterranean Europe included sequences from Italy (North, South, Center), Spain, Portugal, and France. Central Europe encompassed sequences from Germany, Denmark, Romania, Bosnia, Croatia, Bulgaria. Near East comprised sequences from Turkey, Iraq, Iran, Syria, Israel. Africa included sequences from Sudan, Kenya, Morocco, Libya, Mozambique, Egypt, Algeria, Tunisia. East Asia sequences were from Korea, South Korea, Japan, China, Nepal, Mongolia.

The MJ network only including the set of 200 mtDNA sequences generated by us plus the bovine reference sequence V00654 is shown in Fig. 2A. The MJ network showed that most haplotypes were connected to each other in a star like fashion, with a central haplotype (H1) corresponding to the BRS (Acc. No. V00654). Eight haplotypes differed by one mutational event, while the remaining ones differed by two or more mutational events. Each breed showed private haplotypes, sometimes connected to the network through missing intermediate haplotypes (H13, H22, H18, H6). Haplotypes H19 and H4, as well as H6, were the most distant from the central haplotype.

The MJ network including Sardinian, European, Asian and African cattle (Fig. 2B) revealed that about 80% of the Sa cattle sequences belonged to the T3 haplogroup, and 15,8% of sequences shared a specific haplotype belonging to Hg T1. In addition, two haplotypes belonged to Hg T1′2′3. All the SB cattle sequences belonged to Hg T3, except for one haplotype belonging to Hg T1, and one haplotype belonging to Hg T1′2′3, while all SM haplotypes belonged to Hg T3.

The private haplotype belonging to haplogroup T1 has been described in the current work for the first time. This haplotype was characterized by variations at nucleotide positions (np) 16050, 16113 and 16255 typical of Hg T1, and one variation at np 16022, which characterizes the sub-clade termed T1b1^17^. In addition, two other variations (np 15948 and 16136) private to Sardinian native cattle (H6, supplementary Table S2) were detected.

Both haplotypes H4 and H19 (supplementary Table S2) showed a cytosine at np 16255 (typical of Hg T1) but at np 16050 and 16113 they harboured C and T, respectively, as in Hg T3. The nucleotide combination at positions 16050, 16113 and 16255 displayed by haplotypes H4 and H19 was the same one found in haplotype T1′2′3 (Acc. No. EU177840), which is considered a common ancestor of the three T1, T2 and T3 haplogroups^15^. In addition to these nucleotide positions characteristic of the T1′2′3 haplotype, H19 shared with H4 the G>A variation at np 15825, which had been previously reported in only one subject belonging to an unidentified breed^18^, while H4 had an additional rare variation at np 15915, reported only for a subject of the Chianina breed, but within a different haplotype^18^. We detected haplotype H4 in four Sa and one SB cattle distributed in three sampling sites from North East Sardinia (Oschiri and Trinità d'Agultu), while H19 was identified in the Sa breed in just one sampling site (Orotelli) located in the mountains of Central Sardinia.

## Discussion

The hypervariable region of mtDNA was analysed to obtain information about genetic diversity of three local cattle breeds, namely Sarda (Sa), Sardo Bruna (SB) and Sardo Modicana (SM), reared in the island of Sardinia. The F_ST_ analysis revealed a remarkable degree of differentiation between SM and SB. Besides, AMOVA was highly significant, revealing a differentiation between the three breeds. Such genetic differentiation between Sardinian breeds has been also observed by Cesarani et al*.*^13^ and Mastrangelo et al*.*^14^. Cesarani et al*.*^13^ genotyped 19 Sarda, 10 Sardo Bruna and 12 Sardo Modicana cattle with a medium density SNP chip and they revealed that Sardo Modicana individuals cluster close to the Modicana specimens and far apart from Sarda cattle, while Sardo Bruna individuals are placed at an intermediate location between the Brown Swiss and Sarda populations. In an additional study, the genetic diversity of 30 Sarda, 10 Sardo Bruna and 28 Sardo Modicana cattle was investigated with the BovineSNP50 BeadChip^14^. This latter study showed that Sarda animals cluster with Northern and Northern Central Italian breeds^14^. In addition, the lowest F_ST_ value corresponded to the Sarda vs Sardo Bruna pairwise comparison (F_ST_ = 0.016), while Sardo Modicana cattle were more similar to Modicana cattle than to the Sarda ones^14^.

The overall haplotype diversity of Sardinian local cattle (Hd 0.879) was low, when compared to some continental Italian cattle breeds^19^, especially for the SM breed (Hd 0.66). Negative Tajima's D values were calculated for all three breeds (although they were not significant), which might support the hypothesis of a founder effect or a bottleneck^20^. Indeed, the MJ network describing the genetic relationships between the three Sardinian local cattle (Fig. 2A) has a star shaped topology consistent with the occurrence of a single founder effect. This kind of haplotype distribution has already been observed in goats from insular territories^21^. A geographical distribution of major taurine mtDNA haplogroups is reported in supplementary Figure S1.

The MJ network depicted in Fig. 2B illustrates the relationships between the three local breeds of the current study and mtDNA sequences retrieved from public databases which represent, North Africa, Near East, Middle Europe and Mediterranean Europe. The MJ network showed that most of Sardinian samples belonged to Hg T3, as already published for one Sardinian cattle by Achilli et al*.*^15^. Haplogroup T3 has been reported to be the most widespread in South West Europe and originates from the Near East to Europe migration of cattle herds which took place in the Neolithic^15^.

We also detected the presence of one haplotype belonging to Hg T1, and two haplotypes belonging to Hg T1′2′3. The T1 haplotype segregated in both Sa and SB bovines, and it might be private to Sardinian cattle, as a Blast search did not reveal its presence in any other bovine breed. The presence of a private T1 haplotype is consistent with the African introgression of Sardinian cattle breeds, as Hg T1 is representative of African taurine cattle, although Hg T1 has been also identified at low frequencies in continents other than Africa^22^. According to Decker et al*.*^12^, both Iberian and Italian cattle display introgression from African taurine genomes, which probably occurred in two separate events. The Iberian breeds show signatures of a potential introgression from Western African taurine breeds, while several Italian breeds were likely introgressed by East African taurine breeds in which indicine introgression had already occurred^12^.

The presence of *Bos taurus* in Sardinia has been verified in archaeological sites of both Neolithic and Chalcolithic ages, although no zoo-archaeological remains attributable to *Bos primigenius* have been found^23^. It has been reported that in the Neolithic age, maritime routes across the Mediterranean Sea already connected North Africa with Southern Europe^24^. The introduction of African haplotypes into Sardinia might have occurred at that time or later. On the other hand, Sardinia has been historically connected with other territories facing the Mediterranean Sea, from Spain to North Africa, up to present-day Lebanon (Phoenicians), so an indirect African introgression of Sardinian cattle (e.g. through an Iberian intermediary) is also feasible. For instance, zoo-archaeological and molecular studies (mtDNA) conducted in the Sus genus, revealed that pigs were traded between the Italian Peninsula and Sardinia by the end of the second millennium BC (late Bronze age and Iron age) and this gene flow left a genetic signature still detectable in Sardinian feral pigs^25^. During the Bronze Age, the inhabitants of Sardinia were part of the Sea People, who migrated to the Levant at that time, with routes to Sicily and Crete^23,25^.

In conclusion, two hundred and one mtDNA sequences of three Sardinian cattle breeds (Sarda, Sardo Bruna and Sardo Modicana) were analysed in the present investigation. We found a moderate level of haplotype diversity in the Sa and the SB breeds, and low haplotype diversity in the SM. Most haplotypes belonged to haplogroup T3, which is widespread in Europe. In addition, we detected one haplotype belonging to haplogroup T1, and two haplotypes belonging to haplogroup T1′2′3. This T1 haplotype might derive from the African introgression of Sardinian cattle, which might have occurred directly or indirectly.

## Methods

### Sampling and DNA purification

A total of 201 blood samples were collected from Sarda (Sa, n = 131), Sardo Bruna (SB, n = 44), and Sardo Modicana (SM, n = 26) cattle, reared in 16 different areas of Sardinia (supplementary Table S1). In each farm, one to thirty-one female cattle were randomly chosen. Cows were managed under extensive farming systems based on mountainous territories with low agricultural productivity and typically associated with goat farming^9^. DNA was extracted from leukocytes using the Puregene DNA isolation kit (Gentra, Qiagen).

### Mitochondrial DNA analysis

Based on the Bovine Reference Sequence (BRS) GenBank acc. no. V00654^26^, the primer pair MTF: 5'-GACTCAAGGAAGAAACTGC-3' and MTR: 5'-GACTCATCTAGGCATTTTCA-3'^27^ was used to amplify a 1029 bp long segment of the mitochondrial DNA control region, in the nucleotide position range 15,792 – 69 (V00654). Amplification conditions were as follows: 100 ng genomic DNA, 1.5 mM MgCl2, 0·2 mM dNTPs, 1X reaction buffer, 0·2 μM of each primer, and 1-unit Taq DNA polymerase (Platinum, Invitrogen, Life Technologies) in a 25 μl final volume. Thermal protocol was set for an initial denaturation at 94 °C for 2.30 min, and then 35 cycles of 94 °C for 20 s, 56 °C for 30 s, 72 °C for 1.20 min, followed by 72 °C for 5 min were carried out. Amplicons were purified with the ChargeSwitch PCR Clean-Up Kit (Invitrogen, Carlsbad, CA, USA) and then used to perform Sanger sequencing reactions with the BigDye Terminator v3.1 Cycle Sequencing Kit (Applied Biosystems). Sequencing reactions were run in an Applied Biosystems 3730 DNA Analyser (Applied Biosystems, Foster City, CA, USA). Sequencing reactions yielded lower then expected length in many samples, then to make sure that the same fragment is analysed in all individuals, sequences were trimmed to 616 bp. All sequences were submitted to GenBank and given accession numbers KX923119 to KX923319.

### Population genetics analyses

Sequence KX923305 was excluded from the dataset due to an 11 bp deletion. The MEGA version 7.0 software^28^ (https://www.megasoftware.net/) was used to align mtDNA sequences and the DnaSP v.5.10.01 software^29^ (http://www.ub.edu/dnasp/) was employed to estimate nucleotide and haplotype diversities as well as to calculate the F_ST_ coefficients of differentiation according to Hudson et al*.*^30^. The blastN suite of BLAST (http://blast.ncbi.nlm.nih.gov/Blast.cgi) was used to screen the GenBank nucleotide collection database. We limited our search to *Bos taurus* (taxid:9913). Median-Joining (MJ) networks based on mtDNA data were built with the Network v.10 tool^31^ (https://www.fluxus-engineering.com/sharenet.htm). We built a MJ network encompassing 586 sequences, including the 200 Sardinian mtDNA sequences generated by us, plus 386 European, Asian and African cattle mtDNA sequences retrieved from the public databases and representative of all known haplogroups (Hg) (supplementary Table S3)^32–53^. Sequences have been trimmed to obtain an alignment of 487 bp. Polymorphic sites were weighted inversely to the number of mutational events according to Martínez et al*.*^54^. Transversions and transitions were given weights of 3 and 1, respectively. The analysis of molecular variance (AMOVA) was carried out with the Arlequin 3.5 software^55^ (http://cmpg.unibe.ch/software/arlequin35/) and default parameters, while mtDNA haplotype frequencies relative to each sampling location were displayed with the POPART v.1.7 software^56^ (http://popart.otago.ac.nz/index.shtml).

### Ethics statement

The DNA samples used for the present study were extracted from blood samples collected in the context of livestock sanitary programs featured by official veterinarians at local health institutions (Azienda per la Tutela della Salute, ATS) of the Regional Government of Sardinia (Italy), in accordance with relevant guidelines and regulations. All the procedures were approved by the Ethical Animal Care and Experimental Use Committee (Organismo Preposto al Benessere e alla Sperimentazione Animale, OPBSA) of the University of Sassari (protocol number 0122890, approved on 28 September 2021). None of the authors were involved in the collection of the blood samples previously, and just previously collected blood samples were used in this study.

## Supplementary Information

Supplementary Figure S1.

Supplementary Tables.

Supplementary Table S3.

## Data Availability

The original contributions presented in the manuscript are included in the article and Supplementary Material, further inquiries can be directed to the corresponding author.
