# Supplementary material for: Mitochondrial DNA diversity of the Sardinian local cattle stock
Source: Sci Rep. 2022 Feb 15;12:2486. doi: 10.1038/s41598-022-06420-3 (PMC8847569; doi:10.1038/s41598-022-06420-3)
Supplement: Supplementary file 1 — Supplementary Figure S1. [file 41598_2022_6420_MOESM1_ESM.docx]

| 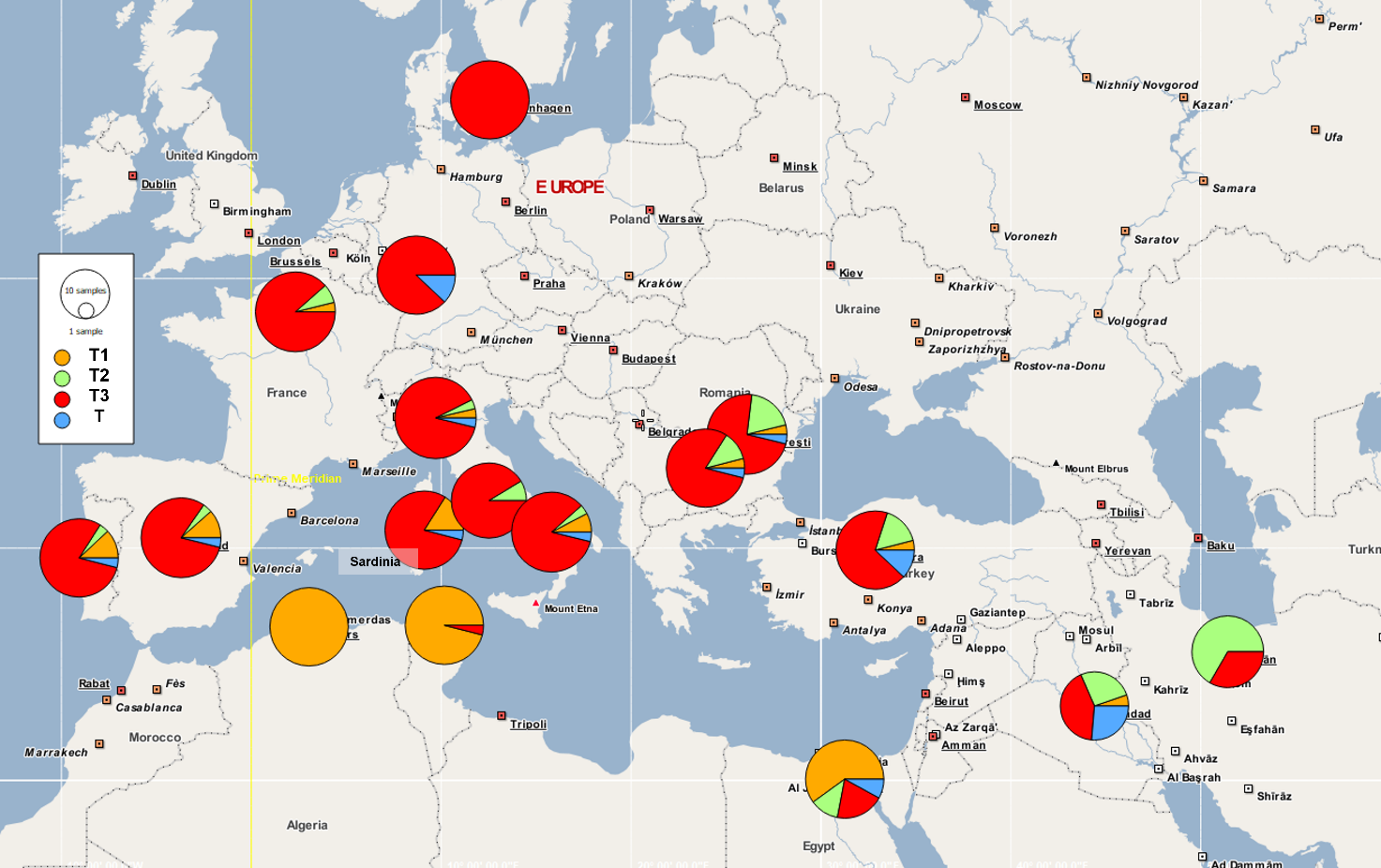 |
| --- |
| **Supplementary Figure S1**. Distribution of the major taurine haplogroups in Europe, North Africa and the Middle East. Haplogroup T included T, T1’2’3 and T5. Haplogroup frequencies were displayed with the POPART v.1.7 software [56] (<http://popart.otago.ac.nz/index.shtml>). |

“Mitochondrial DNA diversity of the Sardinian local cattle stock”

Authors: Petretto, E., Dettori, M.L., Pazzola, M., Manca, F. Amills, M., Vacca, G.M.
