# Supplementary material for: Mitochondrial DNA diversity of the Sardinian local cattle stock
Source: Sci Rep. 2022 Feb 15;12:2486. doi: 10.1038/s41598-022-06420-3 (PMC8847569; doi:10.1038/s41598-022-06420-3)
Supplement: Supplementary file 2 — Supplementary Tables. [file 41598_2022_6420_MOESM2_ESM.pdf]

“Mitochondrial DNA diversity of the Sardinian local cattle stock”

Authors: Petretto, E., Dettori, M.L., Pazzola, M., Manca, F. Amills, M., Vacca, G.M.

Seq ID (isolate)	GenBank Acc.No.	Sampling location	Breed
Seq1_S1	KX923119	Oschiri	Sarda
Seq2_S2	KX923120	Oschiri	Sarda
Seq3_S3	KX923121	Oschiri	Sarda
Seq4_S4	KX923122	Oschiri	Sarda
Seq5_S5	KX923123	Oschiri	Sarda
Seq6_S6	KX923124	Oschiri	Sarda
Seq7_S7	KX923125	Oschiri	Sarda
Seq8_S8	KX923126	Oschiri	Sarda
Seq9_S9	KX923127	Oschiri	Sarda
Seq10_S10	KX923128	Oschiri	Sarda
Seq11_S11	KX923129	Oschiri	Sarda
Seq12_S13	KX923130	Oschiri	Sarda
Seq13_S14	KX923131	Oschiri	Sarda
Seq14_S15	KX923132	Oschiri	Sarda
Seq15_S16	KX923133	Oschiri	Sarda
Seq16_S17	KX923134	Oschiri	Sarda
Seq17_S18	KX923135	Oschiri	Sarda
Seq18_S19	KX923136	Oschiri	Sarda
Seq19_S20	KX923137	Oschiri	Sarda
Seq20_S21	KX923138	Oschiri	Sarda
Seq21_S22	KX923139	Oschiri	Sarda
Seq22_S23	KX923140	Oschiri	Sarda
Seq23_S24	KX923141	Oschiri	Sarda
Seq24_S25	KX923142	Oschiri	Sarda
Seq25_S26	KX923143	Oschiri	Sarda
Seq26_S27	KX923144	Oschiri	Sarda
Seq27_S28	KX923145	Oschiri	Sarda
Seq28_S30	KX923146	Luogosanto	Sarda
Seq29_S31	KX923147	Luogosanto	Sarda
Seq30_S32	KX923148	Luogosanto	Sarda
Seq31_S33	KX923149	Luogosanto	Sarda
Seq32_S34	KX923150	Luogosanto	Sarda
Seq33_S35	KX923151	Luogosanto	Sarda
Seq34_S36	KX923152	Luogosanto	Sarda
Seq35_S37	KX923153	Luogosanto	Sarda
Seq36_S39	KX923154	Luogosanto	Sarda
Seq37_S40	KX923155	Luogosanto	Sarda
Seq38_S41	KX923156	Luogosanto	Sarda
Seq39_S42	KX923157	Luogosanto	Sarda
Seq40_S43	KX923158	Luogosanto	Sarda
Seq41_S44	KX923159	Luogosanto	Sarda
Seq42_S45	KX923160	Luogosanto	Sarda
Seq43_S46	KX923161	Luogosanto	Sarda
Seq44_S47	KX923162	Luogosanto	Sarda
Seq45_S48	KX923163	Luogosanto	Sarda
Seq46_S49	KX923164	Luogosanto	Sarda
Seq47_S50	KX923165	Luogosanto	Sarda
Seq48_S51	KX923166	Orosei	Sarda
Seq49_S52	KX923167	Orosei	Sarda
Seq50_S53	KX923168	Orosei	Sarda
Seq51_S54	KX923169	Orosei	Sarda
Seq52_S55	KX923170	Orosei	Sarda

Seq53_S56	KX923171	Orosei	Sarda
Seq54_S57	KX923172	Orosei	Sarda
Seq55_S58	KX923173	Orosei	Sarda
Seq56_S59	KX923174	Orosei	Sarda
Seq57_S60	KX923175	Orosei	Sarda
Seq58_S61	KX923176	Orosei	Sarda
Seq59_S62	KX923177	Orosei	Sarda
Seq60_S63	KX923178	Orosei	Sarda
Seq61_S64	KX923179	Orosei	Sarda
Seq62_S65	KX923180	Trinità d'Agultu	Sarda
Seq63_S66	KX923181	Trinità d'Agultu	Sarda
Seq64_S68	KX923182	Trinità d'Agultu	Sarda
Seq65_S69	KX923183	Trinità d'Agultu	Sarda
Seq66_S70	KX923184	Trinità d'Agultu	Sarda
Seq67_S72	KX923185	Trinità d'Agultu	Sarda
Seq68_S73	KX923186	Trinità d'Agultu	Sarda
Seq69_S74	KX923187	Trinità d'Agultu	Sarda
Seq70_S75	KX923188	Trinità d'Agultu	Sarda
Seq71_S78	KX923189	Trinità d'Agultu	Sarda
Seq72_S79	KX923190	Trinità d'Agultu	Sarda
Seq73_S80	KX923191	Trinità d'Agultu	Sarda
Seq74_S81	KX923192	Trinità d'Agultu	Sarda
Seq75_S82	KX923193	Trinità d'Agultu	Sarda
Seq76_S83	KX923194	Trinità d'Agultu	Sarda
Seq77_S84	KX923195	Trinità d'Agultu	Sarda
Seq78_S85	KX923196	Trinità d'Agultu	Sarda
Seq79_S86	KX923197	Trinità d'Agultu	Sarda
Seq80_S87	KX923198	Trinità d'Agultu	Sarda
Seq81_S88	KX923199	Trinità d'Agultu	Sarda
Seq82_S89	KX923200	Trinità d'Agultu	Sarda
Seq83_S90	KX923201	Trinità d'Agultu	Sarda
Seq84_S91	KX923202	Trinità d'Agultu	Sarda
Seq85_S92	KX923203	Trinità d'Agultu	Sarda
Seq86_S93	KX923204	Trinità d'Agultu	Sarda
Seq87_S94	KX923205	Trinità d'Agultu	Sarda
Seq88_S95	KX923206	Trinità d'Agultu	Sarda
Seq89_S96	KX923207	Trinità d'Agultu	Sarda
Seq90_S97	KX923208	Trinità d'Agultu	Sarda
Seq91_S98	KX923209	Trinità d'Agultu	Sarda
Seq92_S99	KX923210	Trinità d'Agultu	Sarda
Seq93_Sptz3	KX923211	Villagrande Strisaili	Sarda
seq94_Sptz5	KX923212	Villagrande Strisaili	Sarda
SEq95_Sptz6	KX923213	Villagrande Strisaili	Sarda
Seq96_Sptz7	KX923214	Villagrande Strisaili	Sarda
Seq97_Sptz8	KX923215	Villagrande Strisaili	Sarda
Seq98_Sptz9	KX923216	Orotelli	Sarda
Seq99_Sptz10	KX923217	Alà dei Sardi	Sarda
Seq100_NS1	KX923218	Urzulei	Sarda
Seq101_NS2	KX923219	Urzulei	Sarda
Seq012_NS3	KX923220	Urzulei	Sarda
Seq103_NS4	KX923221	Urzulei	Sarda
Seq014_NS5	KX923222	Urzulei	Sarda
Seq105_NS6	KX923223	Urzulei	Sarda
Seq106_NS7	KX923224	Urzulei	Sarda
Seq107_NS8	KX923225	Urzulei	Sarda
Seq108_NS14	KX923226	Lodè	Sarda
Seq109_NS15	KX923227	Lodè	Sarda
Seq110_NS16	KX923228	Lodè	Sarda

Seq111_NS17	KX923229	Lodè	Sarda
Seq112_NS18	KX923230	Lodè	Sarda
Seq113_NS19	KX923231	Orotelli	Sarda
Seq114_NS20	KX923232	Orotelli	Sarda
Seq115_NS22	KX923233	Orotelli	Sarda
Seq116_NS33	KX923234	Orgosolo	Sarda
Seq117_NS34	KX923235	Orgosolo	Sarda
Seq118_NS35	KX923236	Orgosolo	Sarda
Seq119_NS38	KX923237	Orgosolo	Sarda
Seq120_NS39	KX923238	Orgosolo	Sarda
Seq121_NS40	KX923239	Orgosolo	Sarda
Seq122_NS41	KX923240	Orgosolo	Sarda
Seq123_NS42	KX923241	Orgosolo	Sarda
Seq124_NS43	KX923242	Orgosolo	Sarda
Seq125_NS44	KX923243	Orgosolo	Sarda
Seq126_NS45	KX923244	Orgosolo	Sarda
Seq127_NS47	KX923245	Irgoli	Sarda
Seq128_NS48	KX923246	Irgoli	Sarda
Seq129_NS49	KX923247	Irgoli	Sarda
Seq130_NS51	KX923248	Irgoli	Sarda
Seq131_NS52	KX923249	Irgoli	Sarda
Seq132_SM2	KX923250	Milis	Sardo Modicana
Seq133_SM3	KX923251	Milis	Sardo Modicana
Seq134_SM5	KX923252	Milis	Sardo Modicana
Seq135_SM6	KX923253	Milis	Sardo Modicana
Seq136_SM7	KX923254	Milis	Sardo Modicana
Seq137_SM8	KX923255	Milis	Sardo Modicana
Seq138_SM9	KX923256	Milis	Sardo Modicana
Seq139_SM10	KX923257	Milis	Sardo Modicana
Seq140_SM13	KX923258	Milis	Sardo Modicana
Seq141_SM14	KX923259	Milis	Sardo Modicana
Seq142_SM16	KX923260	Milis	Sardo Modicana
Seq143_SM17	KX923261	Milis	Sardo Modicana
Seq144_SM18	KX923262	Milis	Sardo Modicana
Seq145_SM19	KX923263	Milis	Sardo Modicana
Seq146_SM20	KX923264	Milis	Sardo Modicana
Seq147_SM21	KX923265	Milis	Sardo Modicana
Seq148_SM22	KX923266	Milis	Sardo Modicana
Seq149_SM23	KX923267	Milis	Sardo Modicana
Seq150_SM24	KX923268	Milis	Sardo Modicana
Seq151_SM25	KX923269	Milis	Sardo Modicana
Seq152_SM26	KX923270	Milis	Sardo Modicana
Seq153_SM27	KX923271	Milis	Sardo Modicana
Seq154_SM28	KX923272	Milis	Sardo Modicana
Seq155_SM29	KX923273	Milis	Sardo Modicana
Seq156_SM30	KX923274	Milis	Sardo Modicana
Seq157_SM31	KX923275	Milis	Sardo Modicana
Seq158_SB1	KX923276	Alà dei Sardi	Sardo Bruna
Seq159_SB2	KX923277	Alà dei Sardi	Sardo Bruna
Seq160_SB3	KX923278	Alà dei Sardi	Sardo Bruna
Seq161_SB4	KX923279	Alà dei Sardi	Sardo Bruna
Seq162_SB5	KX923280	Alà dei Sardi	Sardo Bruna
Seq163_SB6	KX923281	Alà dei Sardi	Sardo Bruna
Seq164_SB7	KX923282	Alà dei Sardi	Sardo Bruna
Seq165_SB8	KX923283	Alà dei Sardi	Sardo Bruna
Seq166_SB10	KX923284	Alà dei Sardi	Sardo Bruna
Seq167_SB11	KX923285	Alà dei Sardi	Sardo Bruna
Seq168_SB12	KX923286	Alà dei Sardi	Sardo Bruna

Seq169_SB13	KX923287	Alà dei Sardi	Sardo Bruna
Seq170_SB15	KX923288	Alà dei Sardi	Sardo Bruna
Seq171_SB16	KX923289	Alà dei Sardi	Sardo Bruna
Seq172_SB17	KX923290	Alà dei Sardi	Sardo Bruna
Seq173_SB18	KX923291	Alà dei Sardi	Sardo Bruna
Seq174_SB19	KX923292	Alà dei Sardi	Sardo Bruna
Seq175_SB20	KX923293	Alà dei Sardi	Sardo Bruna
Seq176_SB21	KX923294	Alà dei Sardi	Sardo Bruna
Seq177_SB22	KX923295	Alà dei Sardi	Sardo Bruna
Seq178_SB23	KX923296	Alà dei Sardi	Sardo Bruna
Seq179_SB24	KX923297	Alà dei Sardi	Sardo Bruna
Seq180_SB25	KX923298	Alà dei Sardi	Sardo Bruna
Seq181_SB27	KX923299	Alà dei Sardi	Sardo Bruna
Seq182_SB28	KX923300	Alà dei Sardi	Sardo Bruna
Seq183_SB29	KX923301	Alà dei Sardi	Sardo Bruna
Seq184_SB30	KX923302	Alà dei Sardi	Sardo Bruna
Seq185_SB31	KX923303	Alà dei Sardi	Sardo Bruna
Seq186_SB32	KX923304	Alà dei Sardi	Sardo Bruna
Seq187_NSB9	KX923305	Sedilo	Sardo Bruna
Seq188_NSB10	KX923306	Sedilo	Sardo Bruna
Seq189_NSB13	KX923307	Osidda	Sardo Bruna
Seq190_NSB26	KX923308	Ollolai	Sardo Bruna
Seq191_NSB27	KX923309	Ollolai	Sardo Bruna
Seq192_NSB28	KX923310	Ollolai	Sardo Bruna
Seq193_NSB29	KX923311	Ollolai	Sardo Bruna
Seq194_NSB30	KX923312	Ollolai	Sardo Bruna
Seq195_NSB31	KX923313	Ollolai	Sardo Bruna
Seq196_NSB32	KX923314	Ollolai	Sardo Bruna
Seq197_NSB46	KX923315	Dualchi	Sardo Bruna
Seq198_NSB53	KX923316	Sedilo	Sardo Bruna
Seq199_NSB54	KX923317	Sedilo	Sardo Bruna
Seq200_NSB55	KX923318	Sedilo	Sardo Bruna
Seq201_NSB56	KX923319	Sedilo	Sardo Bruna

Supplementary Table S1. Accession numbers of the mitochondrial hypervariable sequences from Sarda (n = 131), Sardo Bruna (n = 44) and Sardo Modicana (n = 26) cattle.

Np V00654	15825	15915	15926	15943	15948	15952	15960	15965	16022	16050	16055	16058	16068	16081	16085	16113	16119	16122	16126	16133	16136	16138	16150	16185	16200	16228	16247	16248	16255	16260	16264	16301	2	8	21
H1	G	T	T	G	A	A	G	T	G	C	T	C	T	T	T	T	T	T	T	T	A	T	G	G	G	A	C	C	T	C	G	C	C	G	T
T1,2,3	.	.	.	.	.	.	.	.	.	.	.	.	.	.	.	.	.	.	.	.	.	.	.	.	.	.	.	.	C	.	.	.	.	.	.
H2	.	.	.	.	.	.	.	.	.	.	.	.	.	.	.	C	C	.	.	C	.	.	.	.	.	.	.	.	.	.	.	.	.	.	.
H3	.	.	.	.	.	.	.	.	.	.	.	.	.	.	.	C	C	.	.	C	.	.	.	.	.	.	.	.	.	.	.	.	G	.	.
H4	A	C	.	.	.	.	.	.	.	.	.	.	.	.	C	.	.	.	.	.	.	.	.	.	.	.	.	.	C	.	.	T	.	A	.
H5	.	.	.	.	.	.	.	.	.	.	.	.	.	.	.	C	.	.	.	C	.	.	.	.	.	.	.	.	.	.	.	.	.	.	.
**H6**	**.**	**.**	**.**	**.**	**G**	**.**	**.**	**.**	**A**	**T**	**.**	**.**	**.**	**.**	**.**	**C**	**.**	**.**	**.**	**.**	**G**	**.**	**.**	**.**	**.**	**.**	**.**	**.**	**C**	**.**	**.**	**.**	**.**	**.**	**.**
H7	.	.	.	.	.	.	.	.	.	.	.	.	.	.	.	.	.	.	.	C	.	.	.	.	.	.	.	.	.	.	.	.	.	.	.
H8	.	.	.	.	.	.	.	.	.	.	.	.	.	.	.	.	.	.	.	.	.	.	.	.	.	.	.	.	.	.	A	.	.	.	.
H9	.	.	.	.	.	.	.	.	.	.	.	.	C	.	.	.	.	.	.	.	.	.	.	.	.	.	.	.	.	.	.	.	.	.	.
H10	.	.	.	.	.	.	.	.	.	.	.	.	.	.	C	.	.	.	.	.	.	.	.	.	.	.	.	.	.	.	A	.	.	.	.
H11	.	.	.	A	.	.	.	.	.	.	.	.	.	.	.	.	.	.	.	.	.	.	.	.	.	.	.	.	.	.	.	.	.	.	.
H12	.	.	.	.	.	.	.	.	.	.	.	.	.	.	.	C	.	.	.	C	.	.	.	.	.	.	.	.	.	.	.	.	G	.	.
H13	.	.	.	.	.	.	T	C	.	.	.	.	.	.	.	.	.	.	.	.	.	.	.	.	.	.	.	.	.	.	.	.	.	.	.
H14	.	.	.	.	.	.	.	.	.	.	.	.	.	.	.	.	.	.	.	.	.	.	.	.	.	.	.	.	.	.	.	.	.	A	.
H15	.	.	.	.	.	.	.	.	.	.	C	.	.	.	.	.	.	.	.	.	.	.	.	.	.	.	.	.	.	.	A	.	.	.	.
H16	.	.	.	.	.	.	.	.	.	.	.	.	.	.	.	.	.	.	.	.	.	.	A	.	.	.	.	.	.	.	A	.	.	.	.
H17	.	.	.	.	.	.	.	.	.	.	.	.	.	.	.	.	.	.	.	C	.	C	.	.	.	.	.	.	.	.	.	.	.	.	.
H18	.	.	C	.	.	.	.	.	.	.	.	.	.	.	.	.	.	.	.	.	.	.	.	.	.	.	.	.	.	T	.	.	.	.	.
H19	A	.	.	.	.	.	.	.	.	.	.	.	.	.	C	.	.	.	.	.	.	.	.	.	.	.	.	.	C	.	.	.	.	A	.
H20	.	.	.	.	.	G	.	.	.	.	.	.	.	.	.	.	.	.	.	.	.	.	.	.	.	.	.	.	.	.	.	.	.	.	.
H21	.	.	.	.	.	.	.	.	.	.	.	T	.	.	.	.	.	.	.	.	.	.	.	.	.	.	.	.	.	T	.	.	.	.	.
H22	.	.	.	.	.	.	.	C	.	.	.	.	.	.	.	.	.	.	.	.	.	.	.	.	.	.	.	.	.	T	.	.	.	.	.
H23	.	.	.	.	.	.	.	.	.	.	.	.	.	.	.	.	.	C	.	.	.	.	.	.	.	.	.	T	.	.	.	.	.	.	.
H24	.	.	.	.	.	.	.	.	.	.	C	.	.	.	.	.	.	C	.	.	.	.	.	.	.	.	.	T	.	.	.	.	.	.	.
H25	.	.	.	.	.	.	.	.	.	.	.	.	.	G	.	.	.	.	.	.	.	.	.	A	.	.	.	.	.	.	.	.	.	.	.
H26	.	.	.	.	.	.	.	.	.	.	.	.	.	.	.	.	.	.	.	.	.	.	.	.	.	G	.	.	.	.	.	.	.	.	.
H27	.	.	.	.	.	.	.	.	.	.	.	.	.	.	.	.	.	.	.	C	.	.	.	.	.	.	.	.	.	.	A	.	.	.	.
H28	.	.	.	.	.	.	.	.	.	.	.	T	.	.	.	.	.	.	.	.	.	.	.	.	.	.	.	.	.	.	.	.	.	.	.
H29	.	.	.	.	.	.	.	.	.	.	.	.	.	.	.	.	.	.	C	.	.	.	.	.	.	.	.	.	.	T	.	.	.	.	.
H30	.	.	.	.	.	.	.	.	.	.	.	.	.	.	.	.	.	.	.	.	.	.	.	.	A	.	T	.	.	.	.	T	.	.	.
H31	.	.	.	.	.	.	.	.	.	.	.	.	.	.	.	.	.	.	C	.	.	.	.	.	.	.	.	.	.	T	.	T	.	.	.
H32	.	.	.	.	.	.	.	.	.	T	.	T	.	.	.	.	.	.	.	.	.	.	.	.	.	.	.	.	.	.	.	.	.	.	.

Supplementary Table S2. Mitochondrial hypervariable region haplotypes segregating in Sarda, Sardo Bruna and Sardo Modicana cattle (Bovine Reference Sequence GenBank V00654). Np V00654, nucleotide positions related to Accession Number V00654. H1 to H32, haplotypes obtained after alignment of 200 sequences from Sa, SM and SB cattle with DnaSP v.5.10.01 software (Librado and Rozas[29]). In bold, haplotype H6, which was the only one haplotype belonging to T1 Haplogroup in the Sardinian population under study.
